# Resources to Support Families Navigating Early-Stage Type 1 Diabetes: Protocol for a Scoping Review

**DOI:** 10.2196/95168

**Published:** 2026-09-03

**Authors:** Rebecca Anne Pedruzzi, Anna Boggiss, Sarah Black, Keely Bebbington, Carolynn L Smith, Alexandra Tully, Leanne Cromb, Craig Taplin, Yvonne Zurynski, Elizabeth Davis, Aveni Haynes

**Affiliations:** 1 Children’s Diabetes Centre, The Kids Research Institute Australia, 15 Hospital Avenue, Nedlands, Western Australia, 6009, Australia, +61 864564622; 2 Centre for Child Health Research, The University of Western Australia, Perth, Western Australia, Australia; 3 Department of Endocrinology and Diabetes, Perth Children's Hospital, Perth, Western Australia, Australia; 4 Centre for Healthcare Resilience and Implementation Science, Australian Institute of Health Innovation, Macquarie University, Sydney, New South Wales, Australia; 5 Paediatrics, UWA Medical School, The University of Western Australia, Perth, Western Australia, Australia

**Keywords:** early-stage type 1 diabetes, presymptomatic type 1 diabetes, type 1 diabetes, online health information, scoping review, educational resources, youth, families

## Abstract

**Background:**

Advances in the understanding of type 1 diabetes (T1D) natural history have enabled screening for islet autoantibodies and the detection of presymptomatic (early-stage) T1D prior to clinical presentation and the need for insulin replacement therapy. As screening programs expand, increasing numbers of children are being identified with early-stage T1D, creating a pressing need for relevant, accessible, evidence-based educational resources. Due to the rapidly changing paradigm of T1D care in such settings, many health care professionals currently lack awareness or guidance on which educational resources to recommend to families. To date, no review has systematically identified and evaluated educational resources for families navigating early-stage T1D or for health care professionals supporting them.

**Objective:**

This scoping review aims to (1) systematically identify and evaluate educational resources for early-stage T1D; (2) assess resource readability, understandability, quality, and credibility; (3) create an inventory of resources consistent with current care and health literacy guidelines; and (4) identify resource gaps and priorities for future development.

**Methods:**

A comprehensive search of peer-reviewed (MEDLINE, CINAHL, PsycInfo, and Scopus) and gray literature databases (Google advanced searches and targeted searches of professional organizations, advocacy groups, research consortiums, and screening programs) will be conducted to identify English-language resources (eg, fact sheets, websites, videos, and infographics). Resources must target, or be suitable for adaptation for, audiences including youth (aged <25 years), families, parents, carers, and/or health care professionals. Resources must provide information about early-stage T1D, monitoring of early-stage T1D, trajectories, psychosocial aspects, or care navigation. Eligible resources will undergo standardized assessment using validated tools for readability (Flesch-Kincaid Grade Level [FKGL] and Simple Measure of Gobbledygook [SMOG]), understandability (Patient Education Materials Assessment Tool [PEMAT]), information quality (Quality Evaluation Scoring Tool [QUEST]), and credibility (adherence to current early-stage T1D guidelines). Two reviewers will abstract data using a charting tool prepared for the study.

**Results:**

Resources will be grouped by type, audience, and origin. Readability, understandability, quality, and credibility assessments will be presented in tables. Resource gaps will be identified to make recommendations for future resource development. The review will begin in March 2026, and data extraction and analysis will be completed by August 2026, with manuscript submission by October 2026.

**Conclusions:**

This paper presents the protocol for the first scoping review to systematically compile and appraise educational resources for early-stage T1D. The findings will offer a clear overview of the current resource landscape, identify gaps, highlight resources that adhere to current early-stage T1D and health literacy guidelines, and provide a foundation for future development and co-design of fit-for-purpose materials.

## Introduction

### Background

Type 1 diabetes (T1D) is a significant health challenge, affecting more than 1 million young people globally [1], with approximately 140,000 cases reported in Australia alone [2]. T1D is a progressive autoimmune condition defined by the destruction of insulin-producing beta cells in the pancreas [3]. Despite advances in care, T1D continues to be associated with significant morbidity and mortality [4,5], including acute complications such as diabetic ketoacidosis and long-term complications affecting both physical [6] and mental health [7]. Beyond these impacts, T1D imposes substantial psychosocial and economic burdens on families, the health care system, and the broader community [7,8]. Families must navigate complex treatment regimens and the psychological impact of a lifelong chronic condition. The rapid evolution of T1D care models further complicates these challenges, making it essential for both families and health care professionals to access up-to-date information that reflects these changes. Consequently, educational resources must keep pace with the evolving landscape of diabetes management to empower families with the knowledge and skills they need to effectively navigate the complexities associated with T1D.

For example, ongoing advances in the understanding of the pathophysiology of T1D have enabled the identification of individuals with presymptomatic (early-stage) T1D through screening for islet autoantibodies [9-13]. Early-stage T1D is defined by the presence of 2 or more persistent islet autoantibodies with normoglycemia (stage 1) or dysglycemia (stage 2) and precedes symptomatic stage 3, when diagnostic criteria for diabetes are met and insulin therapy becomes necessary [11,14]. T1D is often diagnosed in childhood; hence, there has been a growing international focus on screening during infancy, with Italy being the first country to mandate nationwide screening for children and adolescents aged 1 through 17 years [12,15]. Identifying individuals with early-stage T1D provides opportunities for proactive education and support, monitoring of disease progression, and timely access to clinical trials or disease-modifying therapies [16], as well as preparation for clinical onset. Early detection also opens avenues for preventing acute and long-term complications, supporting psychological readiness, and reducing risks associated with delayed diagnosis, such as diabetic ketoacidosis [16]. However, the benefits of early detection and emerging therapies are contingent upon families and youth having access to reliable, credible, and understandable information.

The ability to find, read, and use health information, along with environmental factors such as supporting policies and practices within the health system [17], are collectively known as health literacy. International health literacy guidelines [18-20] recommend that health resources use plain language; be brief and direct; and use clear headings, bullet points, and illustrations or video content. Additionally, guidelines for readability suggest a grade level between grades 6 [20] and 8 [18]. Health literacy is also affected by environmental factors and personal stressors [21]. Evidence from the broader health screening literature and a recent narrative review of psychosocial considerations for those navigating early-stage T1D demonstrates that individuals’ responses to information are shaped by their personal values, prior experiences with T1D, sociocultural context, and existing tolerance for uncertainty [22]. Parents of children with early-stage T1D commonly experience distress, anxiety, and uncertainty [23,24], particularly when faced with unfamiliar terminology, unpredictable timelines, and evolving clinical guidance. High-quality, trustworthy educational resources supported by consistent, evidence-informed communication by health care professionals are essential to supporting informed, autonomous decision-making and facilitating psychosocial adjustment for families and youth with early-stage T1D [22]. Additionally, quality patient-health care professional relationships are central to enabling families to interpret evolving evidence, navigate uncertainty, and integrate diabetes management tasks into daily life [25].

In the context of early-stage T1D—a rapidly advancing field marked by rapidly evolving knowledge and emerging therapies—both health care professionals and families need information that is accurate, current, and acknowledges the psychosocial considerations of families adjusting to the uncertainties and evolving demands of early-stage T1D [22]. Health care professionals must remain well-informed to deliver consistent, evidence-based advice and resources, and families, who increasingly seek information independently, require information that is aligned with both best practice and lived experience. To date, no review has collated and assessed educational resources currently available for families and youth navigating early-stage T1D.

### Study Objectives

In light of the identified knowledge gap, the scoping review will (1) systematically identify and document early-stage T1D resources available for youth (aged <25 years) and their families and health care professionals supporting youth and their families; (2) assess the quality, credibility, understandability, and readability of materials; (3) create an inventory of resources endorsed as meeting current care guidelines to facilitate future consultation with families and health care professionals; and (4) identify resource gaps and priorities for future development.

## Methods

### Study Design

This scoping review will follow the JBI methodology for scoping reviews [26], structuring the review process into 6 stages, as described below. The scoping review protocol was registered on the Open Science Framework on February 12, 2026, and will be reported according to the PRISMA-ScR (Preferred Reporting Items for Systematic Reviews and Meta-Analyses extension for Scoping Reviews) checklist [27].

### Stage 1: Research Questions

The primary research question of this review is: “What resources are available for youth, families, and health care professionals in understanding and navigating early-stage T1D?” Secondary research questions include: “What is the quality of existing resources?” “Who is the target audience for available resources?” and “What gaps exist in the identified resources currently available?”

### Stage 2: Identifying Relevant Resources

#### Eligibility Criteria

The eligibility criteria for this scoping review are summarized in Textbox 1.

Textbox 1. **Eligibility criteria.**

**Population**

- Primary inclusion—resources available for youth (aged <25 years), families, carers, and parents who support youth aged <25 years
- Secondary inclusion—resources available for health care professionals who support youth and their families

**Concept**

- Early-stage type 1 diabetes (T1D) educational resources that provide information about early-stage T1D, monitoring, trajectories, psychosocial aspects, or care navigation

**Context**

- Resource is available in English
- No date restrictions
- Publicly accessible (free or shareable)

#### Resource Types

Eligible resources include information-based materials such as fact sheets, websites, videos, infographics, webinars, booklets, educational modules, and pamphlets. Resources located via primary research studies that report on the design or evaluation of resources will also be included, as will educational resources used in early-stage T1D clinical trials.

#### Search Strategy

The search strategy was developed in consultation with a skilled medical librarian. Gray literature searches will include Google, professional and advocacy organizations, research consortiums, clinical trial networks, and screening initiatives (Multimedia Appendix 1). A peer-reviewed literature search will be conducted to identify additional resources. This search will be conducted using MEDLINE, CINAHL, PsycInfo, and Scopus databases with no restrictions on study type. Search approaches will be adapted to the functionality of each database, with a detailed search log to record search terms, the date of each search, and the search approach used. All searches will combine terms related to (1) T1D, (2) early stage, (3) biomarker or risk, (4) target audience, and (5) educational resources (refer to Multimedia Appendix 2 for examples).

For databases with advanced search capabilities, including MEDLINE and Google, comprehensive Boolean search strings will comprise [Type 1 Diabetes Terms] AND [Early-Stage Terms] AND [Resource terms] AND [Audience terms] AND [Resource terms] NOT [Type 2 diabetes]. MEDLINE MeSH headings will be used (Multimedia Appendix 3). For organizational websites with limited search functionality, concise search phrases will be used, such as “early stage type 1 diabetes resources” or “stage 1 diabetes education.” For websites without search functions, systematic navigation through relevant sections (eg, “Resources,” “For Families,” and “Education”) will be conducted to identify eligible resources. The following limits will be used: English language and humans. Restricting inclusion to English-language materials ensures feasibility of the review and consistency in data extraction and quality assessment; however, multilingual availability will be recorded during data charting. Key stakeholders, specifically health care staff who deliver early-stage T1D care or who are involved in early-stage T1D surveillance, will be contacted via newsletters, interest groups, and snowball sampling to provide links to resources within their services.

### Stage 3: Selecting Resources

#### Inclusion and Exclusion Criteria

Resources will not be limited to a specific population, geographical location, or health care setting. Resources will be included if they are published in English and provide information about early-stage T1D, monitoring, trajectories, psychosocial aspects, or care navigation. Resources will be excluded if they do not fit the conceptual framework of the study or if they focus on diagnosed T1D (symptomatic stage 3 T1D). Detailed inclusion and exclusion criteria are described in Table 1. Systematic reviews will be excluded, labeled as reviews throughout the screening process, and their reference lists will be reviewed manually for potential omissions.

**Table 1.** Detailed inclusion and exclusion criteria.

	Inclusion	Exclusion
Availability	Must be publicly available or able to be shared by resource creators	Resources that are not publicly available or obtainable from the creator
Topic or focus	Focus of content should be early-stage T1D^a^	Focus of content is insulin-requiring T1D (specifically stage 3 T1D) or other diabetes types
Group for whom the resource is designed	The resource must target, or be suitable for adaptation for, audiences including youth (aged <25 years), families, carers, parents, and/or health care professionals	Resources not suitable for adaptation
Aim or objective	The resource provides information about early-stage T1D, monitoring of early-stage T1D, trajectories, psychosocial aspects, or care navigation.	Study, document, or link does not have any mention of education or resources or supporting information for early-stage T1D.
Language	Resource should be published in English	Resource is only available in a language other than English
Time frame	From inception of the database	None
Resource development location	Any geographic location	None

a T1D: type 1 diabetes.

#### Screening

Resources identified through gray literature searches will be collated into a structured Microsoft Excel–based screening tool to accommodate the diverse resource types, such as websites, fact sheets, infographics, and videos. A screening process will be performed independently by 2 researchers (RAP and AB) for a subset of resources (~10%), with a ≥90% agreement threshold required before proceeding to screening by a single researcher.

For the peer-reviewed literature, reference management software will be used to remove duplicates before beginning a 2-stage screening process. Titles and abstracts will be assessed independently by 2 researchers (RAP and AB) against the inclusion and exclusion criteria. A pilot test will be undertaken on 50 titles and abstracts to assess reviewer agreement. Adjustments to the inclusion criteria may be warranted, if deemed necessary, to ensure consistent interpretation and application of the criteria. The reviewers will then independently screen the remaining titles and abstracts. Any unresolved conflicts will be adjudicated by a third reviewer (SB). Full-text articles will be retrieved for titles and abstracts considered relevant.

Full-text screening will be independently undertaken by the same reviewers, and a pilot test of 10 full-text articles will be performed to assess reviewer agreement. Disagreements will be resolved by discussion or by a third reviewer if necessary.

### Stage 4: Charting the Data

#### Overview

Two reviewers will abstract the data using a charting tool to record the following: (1) resource type or format (eg, video, infographic, fact sheet, booklet, and webinar), (2) source (eg, professional organization, screening initiative, research consortium, advocacy group, and health care provider), (3) platform or delivery channel where relevant (eg, website and hospital intranet), (4) intended audience (family-facing, health care professional–facing, and dual-purpose), (5) geographic origin (eg, Australasian, American, and European), (6) language availability (noting where other languages are available beyond English), (7) stage focus (stage 1, stage 2, and general early-stage), and (8) the quality assessment measures described below. Charting will first begin with a pilot test of 10 articles using the data charting tool. The data charting tool may evolve as reviewers become familiar with the data or identify charting inconsistencies. For example, additional data categories may be needed to answer the research questions.

#### Quality Assessment Measures

The following assessments will be undertaken to determine whether resources are aligned with health literacy guidelines and current early-stage T1D care guidelines (refer to Table 2 for an overview).

**Table 2.** Overview of quality assessment measures by resource type.

Resource type	Health literacy			Fidelity
	Readability: SMOG^a^ via SHeLL^b^ and FKGL^c^	Understandability: PEMAT^d^	Quality: QUEST^e^	Credibility: aligned with early-stage T1D^f^ guidelines
Printable material (text based)	✓	✓	✓	✓
Audiovisual material		✓	✓	✓

a SMOG: Simple Measure of Gobbledygook.

b SHeLL: Sydney Health Literacy Lab.

c FKGL: Flesch-Kincaid Grade Level.

d PEMAT: Patient Education Materials Assessment Tool.

e QUEST: Quality Evaluation Scoring Tool.

f T1D: type 1 diabetes.

#### Readability

Printable materials will be assessed for readability using the Flesch-Kincaid Grade Level (FKGL) [28] and the Simple Measure of Gobbledygook (SMOG) index [29]. Higher FKGL and SMOG scores indicate greater educational requirements for understanding the material. For online health information, information is recommended to be written at a grade level between grades 6 [20] and 8 [18,28,29]. FKGL and SMOG will both be used as they evaluate different linguistic features and together provide a more accurate and reliable estimate of readability for health materials [30], with scores reported separately.

SMOG statistics will be generated using the Sydney Health Literacy Lab (SHeLL) editor to determine a reading level score. The SHeLL tool also assesses complex language, passive voice, text structure, lexical density and diversity, and person-centered language [31] and was developed specifically for application to health education materials. Text, including bullet points, captions, and headings will be copied and pasted directly into the tool.

#### Understandability

Both printable materials and audiovisual information will be assessed for understandability by 2 authors (RAP and AB) using the corresponding subscale and versions of the Patient Education Materials Assessment Tool (PEMAT) [32]. Understandability is the degree to which a resource can be understood by individuals of diverse backgrounds and with varying levels of health literacy [33]. The understandability subscale includes items such as whether the material uses common, everyday language, defines medical terms, and breaks down complex information into manageable chunks. Each item is scored from 0 (disagree) to 1 (agree), with a not applicable option available. Final scores will be calculated as percentages of “agree” responses among applicable items. Scores above 70% indicate acceptable understandability.

#### Quality of Information

The Quality Evaluation Scoring Tool (QUEST) will be used to assess the quality of online health information, applied to both text-based and audiovisual resources [34], by 2 authors (RAP and AB). Although originally developed for online written information, QUEST evaluates content quality domains across 7 items (eg, authorship, evidence cited, conflicts of interest, currency, complementarity, and tone) that are applicable across formats, including videos. Each item is assigned a score and a specific weighting. The total weighted scores range from 0 to 28, with higher scores indicating higher quality.

#### Credibility

Two members of the study team with experience in the field of early-stage T1D (SB and AH) will also independently answer the following question to assess the accuracy and credibility of the information: Is the information consistent with current early-stage T1D guidelines [35]? Response criteria will be “yes completely,” “partially,” or “no, not at all.” Resources scoring “yes completely” will be considered highly credible, those scoring “partially” as moderately credible, and those scoring “no, not at all” as having low credibility. Disagreements will be resolved through discussion or by a third team member (ED).

The readability assessments (FKGL [28], SMOG index [29], and SHeLL [31] analyses) will be completed by a single reviewer (AB), as these tools follow standardized, automated procedures. In contrast, assessments requiring subjective judgment, including understandability (PEMAT), quality of information (QUEST), and credibility, will be independently conducted by 2 reviewers (AB and RAP), with disagreements resolved through discussion or adjudication by a third reviewer (SB).

### Stage 5: Summarizing Findings

Findings will be reported narratively and visualized, following PRISMA-ScR guidelines [27]. A PRISMA (Preferred Reporting Items for Systematic Reviews and Meta-Analyses) flowchart will be used to detail and collate the results of the final review. Our approach will provide a contextual framework that connects resource types, audience, health literacy, fidelity, and content gaps. Specifically, frequency counts of resources by type (eg, leaflets, websites, and videos), number of studies, population or audience (eg, health care professionals, youth, and families), and resource development location (eg, country) will be undertaken and mapped out for presentation using circular charts, bar charts, or tables as the researchers deem appropriate. Quality assessment findings will be summarized and presented in tables or using illustrations for youth, families, and health care professional audiences. Due to the variety of sources included, content analysis will be used to identify themes from the resources that speak to existing knowledge gaps. These gaps will be cross-referenced with literature on the needs expressed by youth and families managing early-stage T1D. Findings will be stratified by the intended audience (eg, family-focused versus health care professional–focused). Resources that score highly in the quality assessment measures of PEMAT, QUEST, and credibility will be included in a study-compiled inventory of resources identified as being suitable for dissemination to families with early-stage T1D.

### Stage 6: Consultation With Key Stakeholders

To identify relevant resources that may not be available online, we will consult with experts in early-stage T1D clinical care and research through our established partnerships. Professional advocacy organizations, research consortiums and networks, clinical trial networks, and screening groups (see Multimedia Appendix 1) will be contacted via email or phone to ask about other relevant resources, including those that are unpublished. Suggestions and contact details of other relevant individuals or groups will be followed up. To track these activities, a table in Excel will be developed and will include details such as group, date of contact, and outcome.

## Results

This review will produce a comprehensive synthesis of existing educational resources for early-stage T1D, including their formats, audiences, origins, and health literacy characteristics. Readability (FKGL, SMOG, and SHeLL), understandability (PEMAT), quality (QUEST), and credibility assessments will be summarized descriptively and presented in tables and visualizations. A key output will be a curated inventory of resources that meet predefined thresholds for understandability, quality, and credibility.

The review will identify gaps in available resources and priorities for future resource development. This project was funded in February 2025. Pilot searching commenced in December 2025. Following refinement of search terms, formal searching of the primary databases began in April 2026. The number of peer-reviewed articles retrieved across databases after deduplication was 494. It is anticipated that data extraction and analysis will be completed by August 2026, with manuscript submission by October 2026.

## Discussion

### Expected Findings

This scoping review will provide the first comprehensive synthesis of educational resources available to families and youth navigating early-stage T1D. These resources will be consolidated into an accessible, online inventory for families and youth with early-stage T1D and health care professionals providing care. The need for resources and support for families navigating early-stage T1D has been identified across the globe [22,36-38]—dovetailing with the implementation of autoantibody screening and longitudinal monitoring [9-13,15]. By systematically identifying, collating, and evaluating existing resources, this review will generate an inventory of currently available resources evaluated methodically to be of high understandability, quality, and credibility. The inventory will be readily accessible, and future research will facilitate local adaptation via community feedback in Western Australia, with the intention that other jurisdictions may also use the inventory to shape their own consumer-informed resource development. Results from this scoping review will be disseminated globally via publication in a relevant peer-reviewed journal and via conference presentations. Results will also be shared with peak body organizations and advocacy groups globally (e.g. ISPAD, SWEET, and Breakthrough T1D) and locally (ANZSPED, Australian and New Zealand T1D Models of Care Community of Practice, and Perth Children’s Hospital early-stage T1D clinic), including for stakeholder education.

### Strengths and Limitations

A major strength of this review is its inclusive and systematic methodology, with clear translational intent to enable practical use of its findings by families and youth navigating early-stage T1D and health care professionals providing their care. The search strategy spans both published and gray literature, allowing for comprehensive identification of the varied formats in which currently available early-stage T1D information is communicated. The application of multiple validated appraisal tools provides a robust assessment of readability, understandability, quality, and credibility.

Several limitations should also be acknowledged. Restricting the review to English-language resources may exclude culturally and linguistically diverse materials of relevance to non–English-speaking families and youth navigating early-stage T1D and health care professionals. However, we aim to address this by providing links, where available, in languages other than English for resources in the curated inventory. Web-based searches are subject to dynamic algorithms, meaning some resources may be missed despite rigorous search procedures. Given the rapid evolution of early-stage T1D research and clinical care pathways, identified materials may also become outdated quickly. All search terms and strategies will be made publicly available to facilitate updates over time. Finally, although quality and credibility evaluations follow established criteria, some subjectivity is inherent when appraising online educational content. Nonetheless, the review’s methodological approach is designed to yield a comprehensive and transparent synthesis of available resources, with emphasis on producing outputs that can be readily applied to real-world clinical and community contexts. In particular, the inventory will serve as a foundational asset, whereby families, youth, and clinicians can adapt or redevelop resources included in the inventory to meet local cultural, linguistic, and service needs.

### Conclusions

This scoping review will address a critical gap by systematically identifying and methodically evaluating existing educational resources related to early-stage T1D. The findings are anticipated to provide an accessible resource for families and youth navigating early-stage T1D and their health care professionals by generating an online inventory of relevant, high-quality resources. The review will also identify gaps in currently available resources to inform priorities for future development of consistent, clear, and fit-for-purpose educational resources.

## Supplementary material

10.2196/95168 Multimedia Appendix 1 Information sources to be searched.

10.2196/95168 Multimedia Appendix 2 Example search terms to be adapted to each platform’s capabilities.

10.2196/95168 Multimedia Appendix 3 MEDLINE search terms.

10.2196/95168 Peer Review Report 1 Peer review report by Rio Tinto Children Diabetes Center, JDRF Global Centre of Excellence in Diabetes Research (Australia).

## Abbreviations

FKGL: Flesch-Kincaid Grade Level
PEMAT: Patient Education Materials Assessment Tool
PRISMA: Preferred Reporting Items for Systematic Reviews and Meta-Analyses
PRISMA-ScR: Preferred Reporting Items for Systematic Reviews and Meta-Analyses extension for Scoping Reviews
QUEST: Quality Evaluation Scoring Tool
SHeLL: Sydney Health Literacy Lab
SMOG: Simple Measure of Gobbledygook
T1D: type 1 diabetes
