# Supplementary material for: Resources to Support Families Navigating Early-Stage Type 1 Diabetes: Protocol for a Scoping Review
Source: JMIR Res Protoc. 2026 Sep 3;15:e95168. doi: 10.2196/95168 (PMC13540619; doi:10.2196/95168)
Supplement: Supplementary file 1 [file resprot-v15-e95168-s001.docx]

### Multimedia Appendix 1 - Information sources to be searched

| Type of information source | Websites to be searched |
| --- | --- |
| General search engines | Google |
| Professional and advocacy organizations | American Diabetes Association (ADA)  Diabetes Australia (DiabetesAU)  Breakthrough T1D Australia (formerly JDRF Australia)  Breakthrough T1D Global (formerly JDRF Global) |
| Research consortiums and networks | Innovative Medicines Initiative for T1D (INNODIA)  Global Platform for the Prevention of Autoimmune (Diabetes GPPAD)  Environmental Determinants of Islet Autoimmunity (ENDIA)  United Kingdom T1D Research Consortium  Australasian Type 1 Diabetes Immunotherapy Collaborative |
| Clinical trial networks | Type 1 Diabetes TrialNet  Bridge T1D |
| Screening programs and studies per region | ADIR (Antibody Detection Israeli Research; Israel)  ASK (Autoimmunity Screening for Kids; United States)  CASCADE (Combined Antibody Screening for Celiac and Diabetes Evaluation; United States)  DAISY (Diabetes Autoimmunity Study in the Young)  DICe (Diabete tipo 1 and Celiacchia; Italy)  DiPiS (Diabetes Prediction in Skåne; Sweden)  DiaUnion (Denmark and Sweden)  Early Check (United States)  EDENT1FI (European Action for the Diagnosis of Early Non-Clinical Type 1 Diabetes for Disease Interception; 8 European Countries)  ELSA (Early Surveillance for Autoimmune Diabetes; United Kingdom)  FR1DA (Früherkennung von Typ-1-Diabetes; Germany)  INNODIA (Innovative Natural History Natural Outreach Development Investigating All aspects of type 1 diabetes; Global)  National Screening Pilot Type 1 Diabetes (Australia)  PLEDGE (Population Level Estimate of Type 1 Diabetes Risk Genes in Children; United States)  SCREENDIA (Screening for Early Detection of Type 1 Diabetes in Spain; Spain)  TEDDY (The Environmental Determinants of Diabetes in the Young; USA/ Europe)  Trial Net Pathway to Prevention (USA, Canada, Europe, Australia)  Type1Screen (Australia and New Zealand) |
| Other research initiatives | T1D Step Ahead  stopT1D  Ask the Experts  INNODIA DETECT |
| Peer-reviewed literature | MEDLINE  CINAHL  PsycInfo  Scopus |
