# Supplementary material for: Resources to Support Families Navigating Early-Stage Type 1 Diabetes: Protocol for a Scoping Review
Source: JMIR Res Protoc. 2026 Sep 3;15:e95168. doi: 10.2196/95168 (PMC13540619; doi:10.2196/95168)
Supplement: Supplementary file 2 [file resprot-v15-e95168-s002.docx]

### Multimedia Appendix 2 – Example search terms to be adapted to each platform’s capabilities

| Term category | Search terms |
| --- | --- |
| Type 1 diabetes | Type 1 diabetes OR T1D or Diabetes Mellitus, type 1 or IDDM or insulin-dependent diabetes mellitus or insulin dependent diabetes mellitus or type 1 Diabet* or juvenile onset diabetes mellitus or autoimmune diabetes |
| Early-stage | early stage OR early-stage OR early onset OR early-onset OR pre-symptomatic OR presymptomatic OR pre-clinical OR preclinical OR incipient OR sub-clinical OR subclinical OR asymptomatic OR stage 1 OR stage 2 OR stage 3 OR stage* |
| Biomarker and risk | autoantibody positiv* OR islet autoantibodies OR multiple islet autoantibodies OR single islet autoantibody OR antibody positiv* OR islet antibodies OR multiple islet antibodies OR single islet antibody GAD antibody OR GADA OR IA-2 antibody OR IA-2A OR IAA OR insulin autoantibodies OR ZnT8 antibody OR ZnT8A OR autoimmun* OR prediction OR risk OR risk score OR genetic risk score OR screening OR positive status OR HLA typing OR Human Leukocyte Antigen typing |
| Youth audience | youth OR adolescent* OR teen* OR child* OR pediatric* OR paediatric* OR young* OR juvenile OR infant OR toddler OR preschool OR student OR patient* OR person* OR individual* OR newly diagnosed |
| Family audience | famil* OR parent* OR caregiver* OR carer* OR guardian* OR sibling* |
| Community audience | peer* OR friend* OR communit* OR school* OR teacher* OR coach OR support person |
| Healthcare professional audience | health* professional* OR stakeholder* OR policy maker* OR policymaker OR general practitioner OR GP OR primary care OR physician* OR psychologist* OR researcher* OR clinician* OR endocrinologist* OR diabetes educator* OR diabetes nurse* OR nurse* OR health* provider OR paediatrician* OR pediatrician* OR social worker* OR dietician* OR dietitian* |
| Resource | guide* OR factsheet OR fact sheet OR information OR booklet* OR video* OR education* OR educational material OR resource* OR leaflet* OR support material* OR toolkit* OR brochure* OR infographic OR webinar OR campaign* OR consensus OR poster* OR module* OR curriculum* OR learning OR handout OR pamphlet OR website OR web page OR web link OR social media |
