# Supplementary material for: Resources to Support Families Navigating Early-Stage Type 1 Diabetes: Protocol for a Scoping Review
Source: JMIR Res Protoc. 2026 Sep 3;15:e95168. doi: 10.2196/95168 (PMC13540619; doi:10.2196/95168)
Supplement: Supplementary file 3 [file resprot-v15-e95168-s003.docx]

### Multimedia Appendix 3 – MEDLINE Search Terms

1. exp Diabetes Mellitus, Type 1/ or (Type 1 diabetes or T1D or Diabetes Mellitus type 1 or IDDM or insulin-dependent diabetes mellitus or insulin dependent diabetes mellitus or type 1 diabet$ or juvenile onset diabetes mellitus or autoimmune diabetes).mp.

2. (exp Early Diagnosis/ or exp Mass Screening/ or exp Asymptomatic Diseases/ or exp Disease Progression/) or (early stage or early-stage or early onset or early-onset or pre-symptomatic or presymptomatic or pre-clinical or preclinical or incipient or sub-clinical or subclinical or asymptomatic or stage 1 or stage 2 or stage 3 or stage$).mp.

3. (exp Autoantibodies/ or exp Biomarkers/ or exp Genetic Predisposition to Disease/ or exp Risk Assessment/ or exp HLA Antigens/ or exp Predictive Value of Tests/) or (autoantibody positiv$ or islet autoantibodies or multiple islet autoantibodies or single islet autoantibody or antibody positiv$ or islet antibodies or multiple islet antibodies or single islet antibody or GAD antibody or GADA or IA-2 antibody or IA-2A or IAA or insulin autoantibodies or ZnT8 antibody or ZnT8A or autoimmun$ or prediction or risk or risk score or genetic risk score or screening or positive status or HLA typing or Human Leukocyte Antigen typing).mp.

4. ((exp Child/ or exp Adolescent/ or exp Infant/ or exp Minors/) or (youth or adolescent$ or teen$ or child$ or pediatric$ or paediatric$ or young$ or juvenile or infant$ or toddler$ or preschool$ or student$ or patient$ or person$ or individual$ or newly diagnosed).mp.) or ((exp Family/ or exp Parents/ or exp Caregivers/ or exp Siblings/ or exp Legal Guardians/) or (famil$ or parent$ or caregiver$ or carer$ or guardian$ or sibling$).mp.) or ((exp Peer Group/ or exp Friends/ or exp Schools/ or exp School Teachers/ or exp Community Networks/) or (peer$ or friend$ or communit$ or school$ or teacher$ or coach or support person).mp.) or ((exp Health Personnel/ or exp Physicians/ or exp Nurses/ or exp Endocrinologists/ or exp Primary Health Care/ or exp General Practitioners/ or exp Psychologists/ or exp Researchers/ or exp Social Workers/ or exp Dietitians/) or (health$ professional$ or stakeholder$ or policy maker$ or policymaker or general practitioner or GP or primary care or physician$ or psychologist$ or researcher$ or clinician$ or endocrinologist$ or diabetes educator$ or diabetes nurse$ or nurse$ or health$ provider or paediatrician$ or pediatrician$ or social worker$ or dietician$ or dietitian$).mp.)

5. (exp Health Education/ or exp Patient Education as Topic/ or exp Educational Materials/ or exp Pamphlets/ or exp Teaching Materials/ or exp Audiovisual Aids/ or exp Social Media/ or exp Internet/ or exp Consumer Health Information/ or exp Information Dissemination/) or (guide$ or factsheet or fact sheet or information or booklet$ or video$ or education$ or educational material or resource$ or leaflet$ or support material$ or toolkit$ or brochure$ or infographic or webinar or campaign$ or consensus or poster$ or module$ or curriculum$ or learning or handout or pamphlet or website or web page or web link or social media).mp.

6. exp Diabetes Mellitus, Type 2/ or (Type 2 diabetes or T2D or T2DM or NIDDM or non-insulin-dependent diabetes or non insulin dependent diabetes).mp.

7. 1 and 2 and 3 and 4 and 5

8. 7 not 6

9. limit 8 to (English language and humans)

Search conducted on 16/12/2025, yielded 440 results.
